# Uncertainty-aware feature mapping and adaptive inference for brain tumor segmentation with missing contrast-enhanced T1-weighted imaging

**DOI:** 10.3389/fnins.2026.1828237

**Published:** 2026-06-17

**Authors:** Weidong Liu, Zhuyin Zhang, Fangfang Deng, Yeyu Xiao

**Affiliations:** 1 Guangzhou University of Chinese Medicine, Guangzhou, China; 2 Department of Radiology, Guangzhou Hospital of Integrated Traditional and Western Medicine, Guangzhou, China

**Keywords:** brain tumor segmentation, deep learning, feature mapping, MRI, test-time adaptation

## Abstract

**Introduction:**

Multi-parametric magnetic resonance imaging (mpMRI) is a cornerstone of clinical brain tumor assessment, yet the contrast-enhanced T1-weighted (T1ce) sequence is frequently unavailable due to contrast-agent contraindications or acquisition failures. Existing segmentation methods degrade substantially when key modalities are missing, motivating the need for robust missing-modality segmentation frameworks.

**Methods:**

We propose UAF-AIMM (Uncertainty-Aware Feature Mapping and Adaptive Inference for Brain Tumor Segmentation with Missing Contrast-Enhanced T1-Weighted Imaging), a 3D brain tumor segmentation framework for the clinically prevalent missing-T1ce scenario. UAF-AIMM integrates three components: (1) MR-Mapper, a cross-modal feature mapping module that recovers T1ce-related semantics in the latent space from observed sequences; (2) Uncertainty-Aware Attention (UAA), which estimates voxel-wise predictive variance and uses learnable gating to fuse features according to their estimated reliability; and (3) Single-Image Test-Time Adaptation (SITA), a per-case calibration strategy that updates only normalization affine parameters to mitigate domain shift at inference.

**Results:**

Evaluated on the BraTS (Brain Tumor Segmentation) 2021 dataset under the missing-T1ce protocol, UAF-AIMM achieves an average Dice similarity coefficient of 89.20% and a 95th percentile Hausdorff distance (HD95) of 5.50 mm, outperforming representative baselines including 3D U-Net, Swin UNETR, mmFormer, and ReCoSeg. The improvement is most pronounced in the contrast-dependent enhancing tumor (ET) subregion.

**Conclusion:**

The results demonstrate that feature-space compensation with explicit reliability control and test-time calibration provides a practical and effective direction for robust brain tumor segmentation under incomplete multi-modal MRI protocols, with potential for translation to heterogeneous clinical environments.

## 1 Introduction

Brain tumors comprise a highly heterogeneous group of central nervous system (CNS) neoplasms. In the World Health Organization (WHO) Classification of Tumors of the CNS, tumor entities and grades are assigned by integrating histopathology with immuno-histochemical and molecular features (Louis et al., 2021). Population-based statistics further indicate that non-malignant tumors are substantially more common than malignant tumors. In the CBTRUS report for tumors diagnosed in the United States (2018–2022), approximately 26.3% of primary brain and other CNS tumors are malignant, whereas 73.7% are non-malignant (Price et al., 2025). Among all histopathological subtypes, meningioma is the most frequent non-malignant entity, accounting for 42.6% of all tumors and 57.4% of non-malignant tumors (Price et al., 2025). For malignant disease, glioblastoma is the single most common diagnosis, representing 13.7% of all tumors and 52.2% of all malignant tumors (Price et al., 2025). More broadly, gliomas constitute the dominant category of primary malignant brain tumors; multiple epidemiological summaries have reported that gliomas account for approximately 80% of malignant primary brain tumors (Schwartzbaum et al., 2006). Beyond primary tumors, brain metastases are frequently encountered in oncology, with an estimated 20% of patients with systemic cancer developing brain metastases over the course of their disease (Achrol et al., 2019).

This etiological and clinical diversity is mirrored by pronounced heterogeneity in imaging phenotype, histopathological characteristics, and molecular profiles, implying that patients may require distinct therapeutic strategies. As a result, accurate, comprehensive, and efficient tumor assessment from medical imaging is essential for risk stratification, treatment planning, and optimized patient management. Meanwhile, rapid advances in artificial intelligence—particularly deep learning—have substantially improved representation learning and pattern recognition, offering a strong methodological foundation for automated medical image analysis. Consequently, the growing integration of AI into clinical workflows (Dorfner et al., 2025; Van Veen et al., 2024; Wong et al., 2024) is increasingly enabling more efficient, accurate, and reproducible approaches for brain tumor detection, segmentation, grading, and the noninvasive prediction of molecular biomarkers.

Among neuro-oncology imaging modalities, multi-parametric magnetic resonance imaging (mpMRI) is a cornerstone of clinical assessment, supporting delineation, volumetric quantification, treatment planning, and follow-up (Achrol et al., 2019; Iv et al., 2018). Conventional brain tumor Magnetic Resonance Imaging (MRI) protocols typically include four complementary sequences: Fluid-Attenuated Inversion Recovery (FLAIR), T2-weighted imaging (T2), pre-contrast T1-weighted imaging (T1), and contrast-enhanced T1-weighted imaging (T1ce). These sequences provide complementary tissue and lesion information. In particular, T2 and FLAIR are sensitive to edema and non-enhancing tumor components, whereas T1ce is critical for identifying blood–brain barrier disruption and active enhancing tumor regions. As such, volumetric analysis of brain tumors from mpMRI constitutes a key quantitative measurement of tumor burden.

Despite the central role of mpMRI, complete multi-sequence acquisition is often unavailable in routine clinical practice. The absence of contrast-enhanced T1-weighted imaging (T1ce) is particularly detrimental: it impairs the delineation of contrast-dependent tumor subregions and, as the critical measurement basis for identifying enhancing tumor (ET) subregions, leads to incomplete information in the MRI measurement system and increased measurement bias (e.g., boundary delineation errors in the ET subregion). Missing modalities may arise from patient-specific factors (e.g., motion corruption, poor compliance, or contraindications to gadolinium-based contrast agents such as severe renal impairment or hypersensitivity) (Blomqvist et al., 2022; Cunningham et al., 2024), as well as system-level factors (e.g., abbreviated protocols for throughput, scanner variability, or corrupted data in retrospective multi-center aggregation). Such modality incompleteness substantially degrades the performance of automated segmentation systems and has motivated extensive research on missing-modality compensation and robust multi-modal representation learning (Lee et al., 2023; Pipoli et al., 2025).

From a modeling perspective, existing solutions for missing-modality brain tumor segmentation can be broadly grouped into four categories: (i) reconstruction-based completion that synthesizes the absent sequence in pixel space, (ii) latent-space feature fusion or completion, (iii) teacher–student knowledge distillation from a full-modality teacher, and (iv) adaptation-based strategies that improve robustness under protocol/site shifts. These approaches have advanced the field, but several limitations remain unresolved in clinically realistic settings. First, image-level synthesis may improve visual plausibility yet does not explicitly optimize task sufficiency for segmentation and can introduce artifacts or registration-sensitive errors. Second, feature-level compensation is often treated deterministically, without explicitly modeling the reliability of compensated representations, allowing compensation errors to be amplified during fusion. This issue is particularly critical when T1ce is missing, because enhancing tumor (ET) delineation strongly depends on contrast-enhanced information. Third, cross-site domain shifts in acquisition protocols, intensity distributions, and artifact patterns further reduce the robustness of fixed inference pipelines.

To address these challenges, we propose *UAF-AIMM* (Uncertainty-Aware Feature Mapping and Adaptive Inference for Brain Tumor Segmentation with Missing Contrast-Enhanced T1-Weighted Imaging), a framework designed for clinically prevalent missing-modality scenarios, with a particular focus on the missing-T1ce setting. Instead of reconstructing the absent modality in pixel space, UAF-AIMM performs *feature-space* compensation by learning a nonlinear cross-modality mapping from the observed sequences to pseudo-T1ce representations. This design aims to recover high-dimensional semantics that are directly useful for downstream segmentation while avoiding explicit image generation.

To reduce the error propagation from imperfect compensation, UAF-AIMM further introduces an uncertainty-aware fusion mechanism that quantifies the reliability of mapped representations and uses uncertainty-modulated attention to reweight feature integration. In this way, high-confidence compensated cues are preserved while low-confidence responses are suppressed, improving boundary consistency and prediction stability, especially for contrast-dependent subregions such as ET. Finally, to improve deployment robustness under a cross-center domain shift, we incorporate a label-free single-case test-time adaptation strategy that performs lightweight test-time calibration using self-supervised signals derived from the predictive distribution, with updates restricted to a small subset of normalization parameters.

In summary, our main contributions are threefold:

(1) *Cross-modality representation mapping for missing-sequence segmentation*. We formulate missing-T1ce compensation as a *feature-space* mapping problem and propose a lightweight Multi-scale Representation Mapper (MR-Mapper) that predicts a pseudo-T1ce representation from available sequences (T1/T2/FLAIR). The mapper is trained to match the semantics of real T1ce features through representation distillation and similarity-preserving constraints, enabling T1ce-aware segmentation *without* pixel-level image synthesis and reducing the risk of anatomically inconsistent hallucinations.
(2) *Uncertainty-aware fusion to control compensation error propagation*. We introduce an uncertainty-aware attention mechanism that explicitly quantifies the reliability of mapped representations and uses uncertainty to modulate multimodal fusion. By suppressing low-confidence pseudo features while preserving informative cues, the proposed fusion improves prediction stability and boundary consistency, particularly for contrast-dependent substructures such as ET.
(3) *Single-case test-time adaptation with constrained updates*. We introduce a per-volume test-time calibration scheme for domain-shifted MRI that requires neither source-domain data nor target labels. Using self-supervised signals from the predictive distribution via entropy minimization, we update only a small subset of test-time parameters (specifically, normalization affine terms), improving robustness and calibration with minimal deployment overhead.

Having outlined the core challenges and our proposed framework, we next review prior literature and related work to situate our approach in the broader context of brain tumor segmentation.

### 1.1 Deep brain tumor segmentation

Radiomics-based neuro-oncology studies have relied on handcrafted descriptors and classical learners, offering interpretability but limited representational capacity and sensitivity to acquisition variability (Breiman, 2001; Cortes and Vapnik, 1995; Patel et al., 2020). Deep CNNs have since dominated volumetric MRI analysis; 3D encoder–decoder architectures in the U-Net family remain the de facto backbones due to their multi-scale representation and dense prediction capability (Milletari et al., 2016). Recent pipelines further improve robustness and accuracy through automated configuration/ensembling and attention mechanisms that suppress background responses and highlight lesion-relevant features (e.g., Attention U-Net) (Oktay et al., 2018). However, most backbones assume a fixed and complete set of input sequences, which are frequently violated in routine clinical practice.

This limitation motivates our use of a strong but practical 3D backbone (Segmentation Residual Network, SegResNet) together with explicit missing-modality compensation and reliability-aware fusion, rather than relying on backbone capacity alone to absorb modality incompleteness.

### 1.2 Missing-modality brain tumor segmentation

Missing modalities arise from protocol heterogeneity, corrupted scans, limited scanner availability, and contrast-agent contraindications. The existing methods fall into three main categories.

#### 1.2.1 Variable-input modeling

HeMIS introduced permutation-invariant fusion by aggregating modality-specific feature statistics (Havaei et al., 2016). Subsequent studies have improved fusion with hybrid or multi-scale designs (e.g., Hi-Net) (Zhou et al., 2020) and enhanced robustness using missing-aware encoders/fusion schemes and modality dropout during training (Feng et al., 2023; Shen and Gao, 2019). These methods are computationally attractive, but their performance is fundamentally bounded by the information content of the remaining modalities. When T1ce is absent, the delineation of enhancing tumor often degrades because enhancement-specific cues are not directly available.

#### 1.2.2 Image-level synthesis

Another direction synthesizes the missing sequence and applies a fixed-input segmenter. Conditional GAN based multi-contrast translation can recover enhancement-like appearance, but may violate anatomical consistency and introduce hallucinated details that mislead boundary prediction (Conte et al., 2021; Dar et al., 2019). Diffusion-based translation has recently been explored to improve stability and generation quality relative to purely adversarial objectives in some settings (Özbey et al., 2023). Nonetheless, optimizing visual fidelity does not guarantee task-sufficient cues for subregion discrimination, especially around ambiguous enhancing regions.

#### 1.2.3 Latent compensation and distillation

Latent-space methods learn modality-invariant representations and can jointly support segmentation and completion, e.g., VAE-based U-HVED (Dorent et al., 2019) and its residual/multi-factor variants such as DRM-VAE (Zhu et al., 2021). Transformer-based fusion further models intra−/inter-modal interactions to capture long-range cross-modal relations, including mmFormer and MMCFormer (Karimijafarbigloo et al., 2024; Zhang et al., 2022), with related clinical multimodal Transformers also reported (Ting and Liu, 2023). A persistent limitation is that compensation is often fused deterministically without explicit reliability control; errors in completion/fusion can be amplified and propagated to final predictions. Knowledge distillation from full-modality teachers has been used to mitigate missing inputs, but it introduces additional training complexity and remains sensitive to teacher quality; recent frameworks emphasize the need to model compensation reliability under real missing patterns (Zhu et al., 2025).

Taken together, prior missing-modality methods establish the value of completion and fusion, but they leave a practical gap in *task-oriented feature compensation with explicit reliability control*. This gap directly motivates our feature-space MR-Mapper and uncertainty-aware fusion (UAA), which aim to recover enhancement-related semantics while suppressing unreliable pseudo cues before decoding.

### 1.3 Domain shift and test-time adaptation

Cross-center deployment introduces domain shifts arising from scanner and protocol variations, intensity distribution changes, and site-specific artifacts. Domain adaptation methods attempt to reduce this gap through distribution alignment and can be combined with missing-modality learning (e.g., ACN) (Wang et al., 2021). However, alignment may become unstable when domain shift co-occurs with a compensation error, which is common in incomplete-modality settings.

Test-time adaptation (TTA) provides a deployment-time alternative by calibrating the model using only test data. TENT, for example, minimizes the prediction entropy while updating only the normalization parameters, enabling source-free adaptation with constrained parameter updates (Wang et al., 2020). Recent medical imaging studies have extended TTA to segmentation and highlighted the need to balance adaptation gains against stability and inference overhead in clinical workflows (Chen et al., 2025; Joshi et al., 2025).

These observations motivated our conservative single-case test-time adaptation design, which restricts updates to normalization affine parameters and is intended to improve robustness under domain shift without substantially compromising deployment practicality.

Organization: The remainder of this paper is organized as follows: Section 2 introduces the proposed Materials and Methods, including the SegResNet backbone, MR-Mapper, UAA, SITA modules, and the multi-stage training strategy; Section 3 describes the experimental setup and reports comparative results, ablation studies, robustness analyses, and cross-dataset generalization; Section 4 provides a comprehensive discussion of clinical relevance, positioning with respect to prior work, empirical gain analysis, interpretability, and limitations; finally, Section 5 concludes the paper.

## 2 Materials and methods

### 2.1 Problem formulation

Each multi-parametric MRI (mpMRI) sample consists of four co-registered volumetric sequences, as defined in Equation 1:


$$X=\{X^{\text{FLAIR}},X^{T1},X^{T1\mathrm{ce}},X^{T2}\},X^{m}\in {\mathbb{R}}^{H\times W\times D}$$ (1)

Where *m* ∈ {FLAIR, T1, T1ce, T2} indexes the imaging modality and *H*, *W*, and *D* denote the spatial dimensions. Given the voxel-wise annotation *Y*, we seek to learn a segmentation model $f_{\theta }$ that predicts tumor subregions (e.g., Whole Tumor (WT), Tumor Core (TC), and Enhancing Tumor (ET)) in a dense manner, as formalized in Equation 2:


$$\hat{Y}=f_{\theta }(X)$$ (2)

We consider the clinically prevalent *missing-T1ce* scenario. During training, all four modalities are available and are used for supervised optimization. At inference time, the contrast-enhanced sequence *X*^T1ce^ is assumed to be missing and is excluded from the input. The model therefore operates on the set of available modalities defined in Equation 3:


$$\begin{array}{c} X_{\text{avail}}=\{X^{m}|m\in \{\;\text{FLAIR},T1,T2\;\}\} \end{array}$$ (3)

The goal is to achieve segmentation performance close to the full-modality setting while relying only on the available sequences at test time and without introducing external data. To this end, we learn a cross-modal feature mapping that recovers T1ce-related semantics in the latent space from $X_{\text{avail}}$ , and perform the final prediction based on the compensated representations.

### 2.2 Overview of the proposed framework

**Figure 1:**
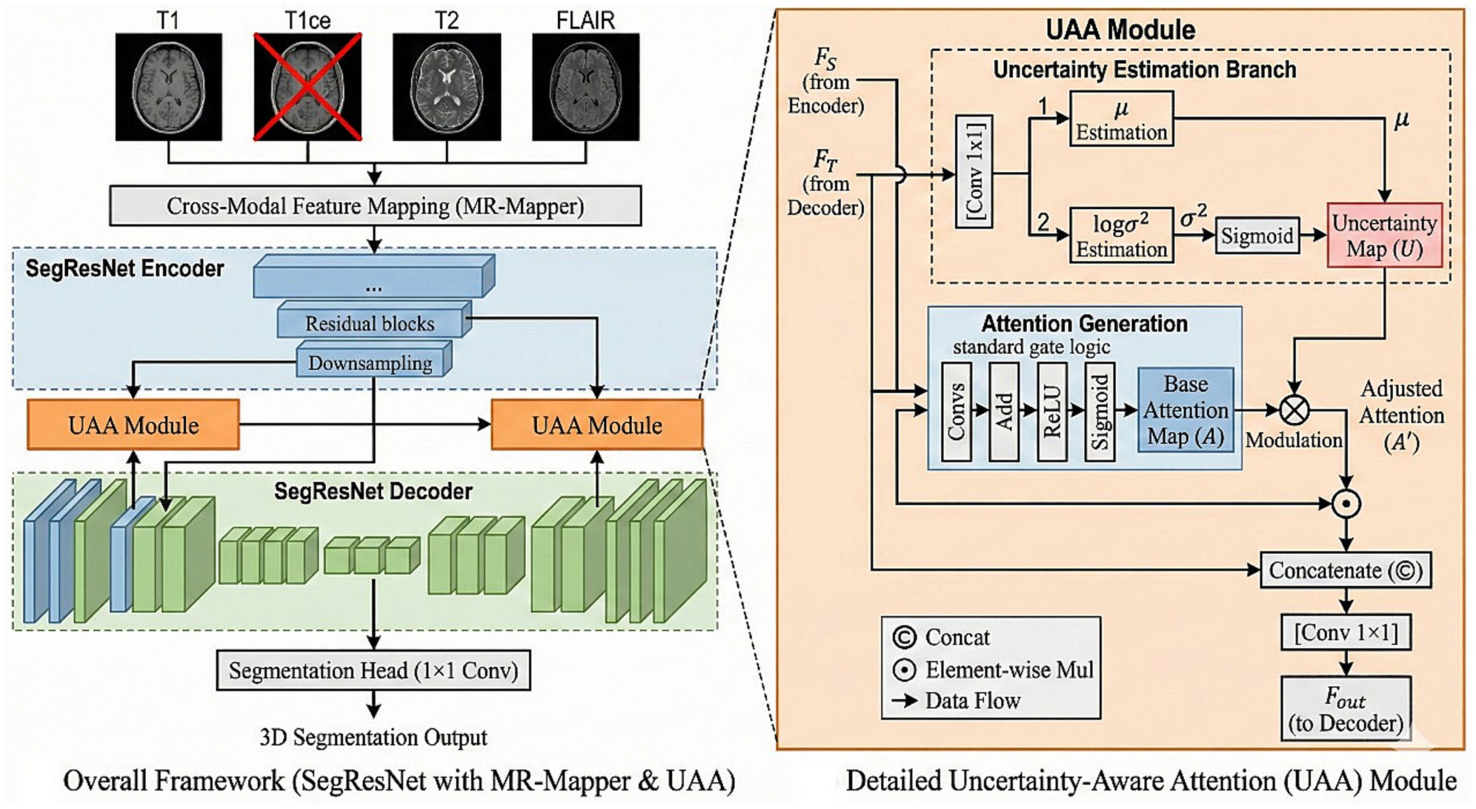
Overall framework of the proposed method based on SegResNet. **(A)** Overall framework (SegResNet with MR-Mapper & UAA). **(B)** Detailed Uncertainty-Aware Attention (UAA) Module.

As shown in Figure 1, our framework builds on SegResNet to perform 3D brain tumor segmentation under the clinically prevalent *missing-T1ce* setting. The proposed network comprises the following three key components: First, a cross-modal feature mapping module (*MR-Mapper*) performs latent-space completion by inferring *pseudo-T1ce* representations from the observed modalities (T1/T2/FLAIR), thereby recovering enhancement-related semantics that would otherwise be unavailable. Second, a SegResNet encoder–decoder backbone extracts multi-scale features through residual blocks with progressive downsampling and restores spatial resolution via hierarchical upsampling, producing dense voxel-wise predictions. Third, an uncertainty-aware attention module (*UAA*) is placed at the encoder–decoder feature interaction stages (two instances in Figure 1A), and the detailed structure of the UAA Module is shown in Figure 1B. The UAA explicitly estimates the reliability of candidate features and uses it to modulate attention during fusion, attenuating low-confidence pseudo cues and reducing error propagation in the decoder.

### 2.3 SegResNet backbone

SegResNet is adopted as the 3D segmentation backbone. The network follows an encoder–decoder architecture: the encoder stacks residual blocks with progressive downsampling to extract multi-scale volumetric representations, and the decoder restores spatial resolution via hierarchical upsampling with skip connections to recover fine structural details. A final 1 × 1 × 1 convolution projects the decoded features to voxel-wise logits for multi-class tumor subregion segmentation. Following the BraTS convention, the predicted subregions include *whole tumor* (WT), *tumor core* (TC), and *enhancing tumor* (ET). To integrate compensated information reliably, we insert UAA modules at two encoder–decoder interaction scales to modulate feature fusion based on the estimated uncertainty of the pseudo features.

### 2.4 MR-mapper: cross-modal feature mapping

The MR-Mapper compensates for the missing T1ce sequence by predicting pseudo-T1ce features in the latent space from the observed modalities. Let { $F_{s}^{T1},F_{s}^{T2},F_{s}^{F\;\text{LAIR}}$ } denote the multi-scale encoder features extracted from {T1, T2, FLAIR} at scale *s*. We learn a nonlinear mapping defined in Equation 4:


$$\begin{array}{c} {\tilde{F}}_{s}^{T1\mathrm{ce}}=M_{\theta ,s}(F_{s}^{T1},F_{s}^{T2},F_{s}^{F\;\text{LAIR}}) \end{array}$$ (4)

Where ${\tilde{F}}_{s}^{T1\mathrm{ce}}$ is the synthesized pseudo-T1ce representation. Concretely, we concatenate the observed-modality features channel-wise and apply a lightweight two-layer 1 × 1 × 1 MLP implemented with pointwise convolutions, as formalized in Equation 5:


$$\begin{array}{c} {\tilde{F}}_{s}^{T1\mathrm{ce}}=\text{IN}({\text{Conv}}^{\left(2\right)}(\text{GELU}({\text{Conv}}^{\left(1\right)}([F_{s}^{T1,F_{s}^{T2,F_{s}^{\text{FLAIR}}}}])))) \end{array}$$ (5)

Where ${\text{Conv}}^{\left(1\right)}$ projects to a hidden width of 64, ${\text{Conv}}^{\left(2\right)}$ maps to the target channel dimension, and IN denotes InstanceNorm3d with learnable affine parameters. We instantiate MR-Mapper at three scales (1/8, 1/4, and 1/2 resolution) and inject ${\tilde{F}}_{s}^{T1\mathrm{ce}}$ into the corresponding encoder–decoder fusion points to provide both coarse enhancement context and fine boundary cues, which are particularly important for delineating the enhancing tumor (ET). The training objectives used to learn ${ℳ}_{\theta ,s}$ (including auxiliary feature supervision when available) are described in Sec.2.7. At inference, ${\tilde{F}}_{s}^{T1\mathrm{ce}}$ is fused with the observed-modality features and decoded to produce the final prediction.

### 2.5 UAA: uncertainty-aware attention

**Figure 2:**
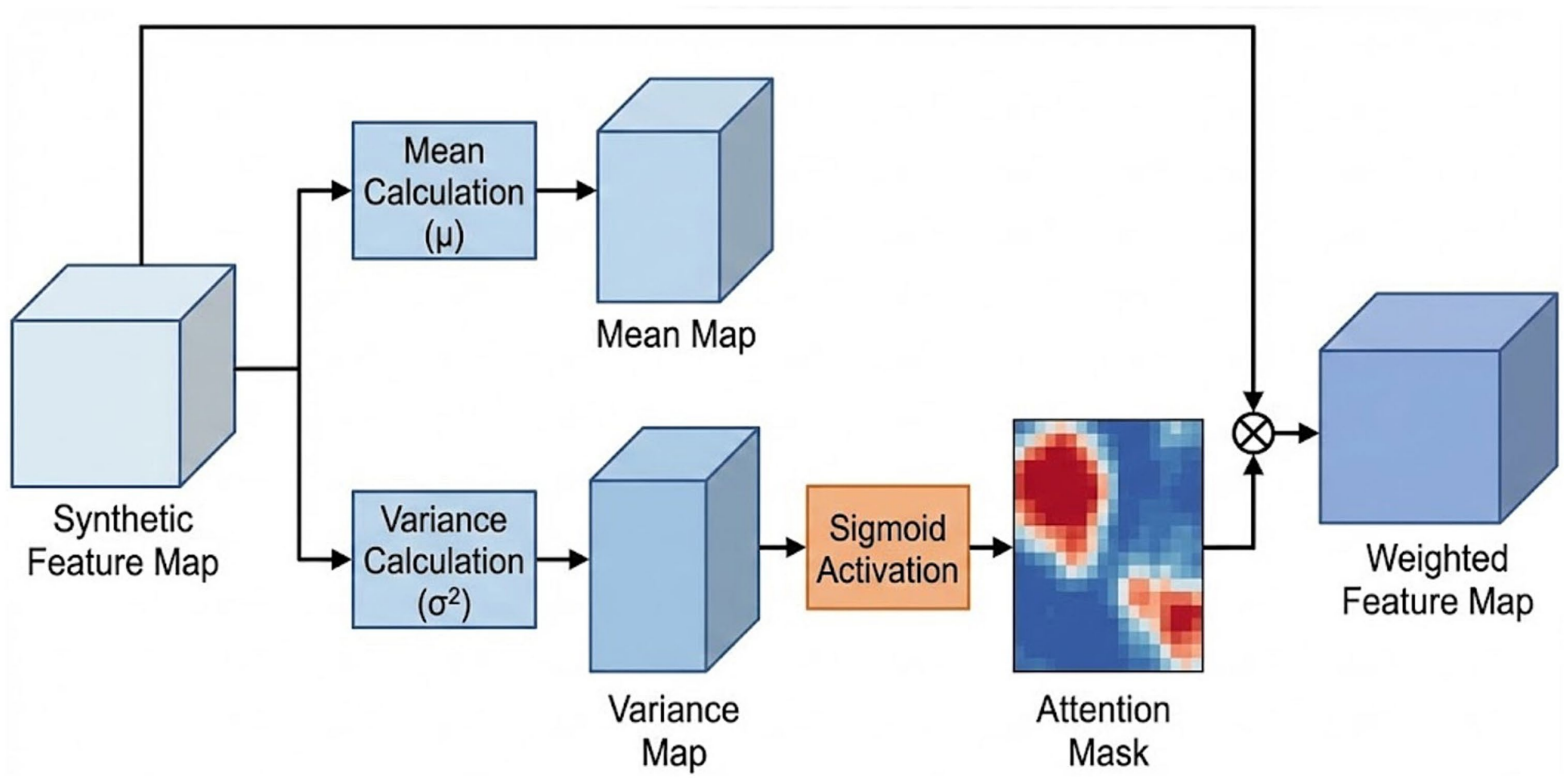
Overview of the uncertainty-aware attention (UAA) mechanism. Given the synthesized feature map, UAA estimates the mean and variance via Monte-Carlo sampling and converts the variance into an attention mask to reweight the synthesized features.

A key challenge in missing-modality segmentation is that the synthesized pseudo-T1ce features are not uniformly reliable across subjects and spatial locations, especially near tumor boundaries and small lesions. Treating synthesized features as equally trustworthy as the observed modalities can inject spurious cues into the decoder and amplify errors (see Figure 2).

To address this issue, we propose a UAA mechanism that assigns a voxel-wise confidence weight to the synthesized features and performs reliability-guided fusion.

#### 2.5.1 Uncertainty estimation for synthesized features

Following a Bayesian approximation via Monte–Carlo (MC) sampling, we characterize the synthesized feature map as a distribution rather than a point estimate. Let $F_{\mathrm{syn}}$ denote the synthesized (pseudo-T1ce) feature map produced by the MR-Mapper. With dropout enabled, we perform *K* (*K* = 10 by default) stochastic forward passes to obtain samples, as denoted in Equation 6:


$$\begin{array}{c} {\left\{F_{\mathrm{syn}}^{\left(k\right)}\right\}}_{k=1}^{K} \end{array}$$ (6)

We compute the mean map and variance map as defined in Equation 7:


$$\begin{array}{c} \mu =\frac{1}{K}\sum_{k=1}^{K}F_{\mathrm{syn}}^{\left(k\right)},\sigma^{2}=\frac{1}{K}\sum_{k=1}^{K}{\left(F_{\mathrm{syn}}^{\left(k\right)}-\mu \right)}^{2} \end{array}$$ (7)

Here, $\sigma^{2}$ quantifies voxel−/channel-wise uncertainty of the synthesized representation, where larger values indicate lower confidence and typically occur around ambiguous boundaries or imaging artifacts. We use the mean map as the synthesized feature representation, as shown in Equation 8:


$$\begin{array}{c} {\tilde{F}}^{T1\mathrm{ce}}=\mu \end{array}$$ (8)

#### 2.5.2 Uncertainty-to-attention mapping

We convert the variance map into a soft gating weight using learnable scaling parameters, as formalized in Equation 9:


$$\begin{array}{c} A=\frac{1}{1+\exp(\alpha \cdot \sigma^{2}+\beta )}=\text{Sigmoid}(-(\alpha \cdot \sigma^{2}+\beta )) \end{array}$$ (9)

Where *α* and *β* are learnable values. This formulation down-weights high-uncertainty regions while preserving reliable synthesized cues. We adopt scalar α and β as learnable parameters shared across all channels and spatial locations to maintain parameter efficiency in the fusion module.

As shown in Table 1, channel-wise and spatial-wise variants yield only marginal performance improvements (<0.2 percentage points in average Dice) at the cost of orders of magnitude more learnable parameters, supporting the sufficiency of the simpler scalar formulation for this setting.

**Table 1:** Comparison of scalar, channel-wise, and spatial-wise variants of the learnable parameters *α* and *β* in the uncertainty-to-attention mapping (Section 2.5.2).

Variant	Extra Params	Avg Dice (%)	HD95 (mm)	Notes
Scalar (default)	+2	89.20	5.50	*α*, *β* shared globally
Channel-wise	+128	89.32	5.48	Per-channel *α*, *β*
Spatial-wise	+256	89.28	5.46	Per-voxel *α*, *β* via 1 × 1 × 1 conv

Results are reported under the missing-T1ce protocol on the BraTS 2021 validation split.

#### 2.5.3 Reliability-guided fusion

Let $F_{\mathrm{obs}}$ denote the feature representation extracted from the observed modalities. UAA fuses $F_{\mathrm{obs}}$ with the uncertainty-weighted synthesized feature as defined in Equation 10:


$$\begin{array}{c} F_{\text{fusion}}=\psi (\text{Concat}(F_{\mathrm{obs}},A⊙{\tilde{F}}^{T1\mathrm{ce}})) \end{array}$$ (10)

Where ⊙ denotes the Hadamard product, Concat (·) concatenates the feature maps along the channel dimension, and ψ (·) is a 1 × 1 × 1 convolution for feature mixing. This design enables the network to selectively exploit high-confidence synthesized semantics while suppressing unreliable regions, thereby achieving a principled balance between information completion and noise injection.

#### 2.5.4 Fusion output

Finally, the adjusted attention is applied to the source feature and fused with the target branch. Following the design in Figure 1B, we concatenate the decoder feature $F_{T}$ with the attended source feature $A^{\prime }⊙F_{S}$ , and use a 1 × 1 × 1 convolution ψ (·) to produce the output feature passed to the decoder, as formalized in Equation 11:


$$\begin{array}{c} F_{\text{out}}=\psi (\text{Concat}(F_{T},A^{\prime }⊙F_{S})) \end{array}$$ (11)

Where ⊙ denotes element-wise multiplication. By jointly leveraging uncertainty estimation and attention modulation, the UAA preserves complementary information while suppressing low-confidence sources, resulting in more stable predictions and improved boundary delineation, especially for the ET region.

### 2.6 SITA: single-image test-time adaptation at inference

**Figure 3:**
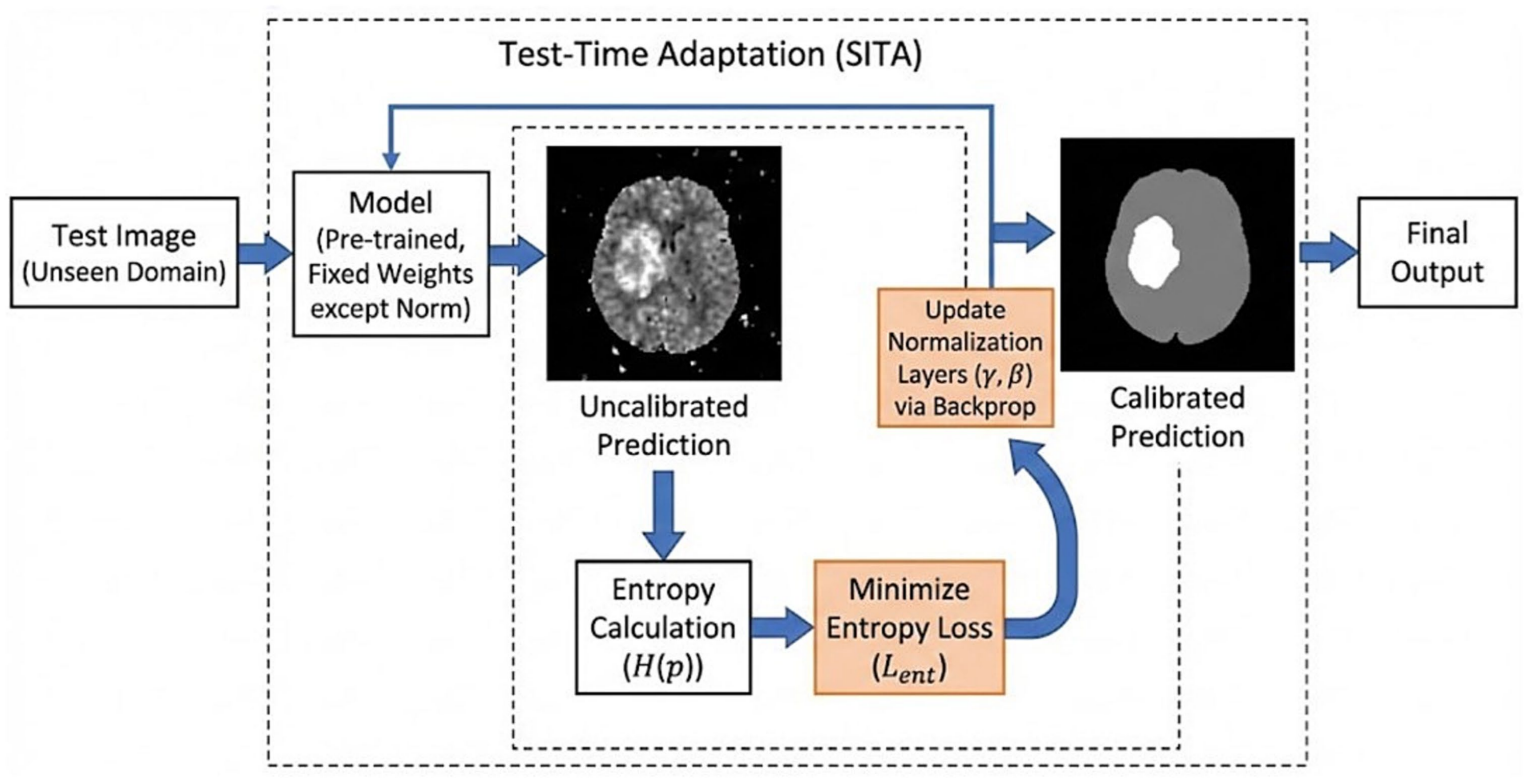
Overview of the proposed Single-Image Test-Time Adaptation at Inference (SITA). Given an unseen-domain test image, the pretrained model first produces an uncalibrated prediction. SITA then minimizes an entropy loss on the prediction to update only the affine parameters (*γ*, *β*) of normalization layers (all convolutional weights are frozen), yielding a calibrated prediction and the final segmentation output.

Supervised segmentation models are commonly trained under the assumption that training and test samples follow the same distribution. In clinical deployment, however, variations in scanner vendors, acquisition protocols, and reconstruction pipelines can induce substantial distribution shifts, leading to degraded performance. To improve the robustness under such heterogeneity, a Single-Image Test-Time Adaptation strategy (SITA) is adopted, which performs lightweight, per-case calibration at inference without requiring target-domain labels (see Figure 3).

The SITA follows a controlled statistics-calibration paradigm. For each test volume *x*, all convolutional weights are frozen, and only the affine parameters (*γ*, *β*) of the normalization layers are updated, thereby constraining the adaptation capacity and reducing the risk of a catastrophic drift. The adaptation is driven by entropy minimization, based on the premise that a better-calibrated model should produce more confidence (lower entropy) predictions on the current test case. The per-case objective is defined in Equation 12:


$$\begin{array}{c} L_{\mathrm{ent}}(x)=-\frac{1}{|\Omega |}\sum_{i\in \Omega }\sum_{c=1}^{C}p_{c,i}(x)\log p_{c,i}(x) \end{array}$$ (12)

Where Ω denotes the voxel set, C is the number of classes, and $p_{c,i}(x)$ is the predicted probability of voxel i belonging to class c . Starting from the pretrained model parameters, we perform a small number of gradient-descent steps on ${ℒ}_{\mathrm{ent}}(x)$ using only the current test volume, and then output the adapted prediction for x . By updating only normalization affine parameters on a per-volume basis, SITA adapts to case-specific intensity characteristics while preserving the learned semantic representations, which helps mitigate cross-site domain shifts in heterogeneous clinical data.

### 2.7 Multi-stage training strategy and objectives

To make the optimization procedure consistent with the deployment condition (i.e., inference without T1ce), a three-phase training and adaptation strategy is adopted. The phases are designed to (i) learn a reliable cross-modal feature mapping using full supervision when available, (ii) train the segmentation model under the missing-T1ce input constraint with reliability-aware fusion, and (iii) perform conservative single-case test-time calibration under domain shift.

#### 2.7.1 Phase 1: feature mapping pre-training

In Phase 1, we pre-train the cross-modal feature mapping module (MR-Mapper) using the full four-modality training data. While the mapper takes only the observed modalities {T1, T2, FLAIR} as inputs, the real T1ce sequence is available and used to provide feature-level supervision. Let ${\tilde{F}}^{T1\mathrm{ce}}$ denote the pseudo-T1ce feature predicted by the MR-Mapper, and $F_{\text{real}}^{T1\mathrm{ce}}$ denote the encoder feature extracted from the real T1ce branch (teacher). We optimize MR-Mapper with a distillation objective combining feature regression and directional alignment, as defined in Equation 13. The teacher features for MR-Mapper pre-training are extracted from a separately pre-trained T1ce encoder branch with identical architecture to the main SegResNet encoder but with independent parameters. This teacher encoder is pre-trained exclusively on T1ce volumes and its weights are frozen throughout both Phase 1 and Phase 2, thereby preventing information leakage from the validation or test distributions.


$$\begin{array}{c} L_{\text{distill}}=\mathrm{MSE}({\tilde{F}}^{T1\mathrm{ce}},F_{\text{real}}^{T1\mathrm{ce}})+\lambda_{\cos}(1-\cos({\tilde{F}}^{T1\mathrm{ce}},F_{\text{real}}^{T1\mathrm{ce}})) \end{array}$$ (13)

Where $\lambda_{\cos}$ = 0.5. This phase is trained for 20 epochs, yielding a mapper that captures enhancement-related semantics in the latent space without requiring pixel-level synthesis.

#### 2.7.2 Phase 2: end-to-end segmentation under missing T1ce

Phase 2 trains the full segmentation pipeline to operate under missing-T1ce inputs. Importantly, the MR-Mapper parameters are *frozen* after Phase 1 to prevent the segmentation loss from destabilizing the learned mapping. During this phase, the model receives only {T1, T2, FLAIR} as input; the T1ce volume is *not* used as an input channel and is only used to compute auxiliary supervision terms.

*Segmentation loss*. Let $p_{c,i}$ denote the predicted probability for voxel i belonging to class c and $g_{c,i}$ ∈ {0, 1} the corresponding one-hot label. We use a multi-class soft Dice loss, as defined in Equation 14:


$$\begin{array}{c} L_{\text{Dice}}=1-\frac{2\sum_{c=1}^{C}\sum_{i\in \Omega }p_{c,i}g_{c,i}+\varepsilon }{\sum_{c=1}^{C}\sum_{i\in \Omega }p_{c,i}+\sum_{c=1}^{C}\sum_{i\in \Omega }g_{c,i}+\varepsilon } \end{array}$$ (14)

Where Ω is the voxel set, C is the number of classes, and ε is a small constant.

*Distillation consistency*. To retain enhancement-related semantics during segmentation training, we optionally keep the distillation term ${ℒ}_{\text{distill}}$ as an auxiliary constraint (computed against real-T1ce teacher features), while still restricting the model input to three modalities.

*Uncertainty regularization*. In addition to using uncertainty to modulate fusion weights in UAA, we further introduce a lightweight uncertainty regularizer to discourage excessively uncertain pseudo features. Concretely, we penalize the mean predicted variance, as formalized in Equation 15:


$$\begin{array}{c} L_{\mathrm{unc}}=\frac{1}{|\Omega |}\sum_{i\in \Omega }\sigma_{i}^{2} \end{array}$$ (15)

Where $\sigma_{i}^{2}$ is the voxel-wise predictive variance estimated in UAA. This term encourages the compensated representations to be confident where possible, while UAA still suppresses unreliable regions through gating.

*Overall objective*. The Phase 2 training objective is defined in Equation 16:


$$\begin{array}{c} L_{\text{total}}=L_{\text{Dice}}+\lambda_{\text{distill}}L_{\text{distill}}+\lambda_{\mathrm{unc}}L_{\mathrm{unc}} \end{array}$$ (16)

Where we set $\lambda_{\text{distill}}$ = 0.1 and $\lambda_{\mathrm{unc}}$ = 0.01 unless otherwise stated.

#### 2.7.3 Phase 3: test-time adaptation (SITA)

Phase 3 is performed *only at inference* to handle domain shifts across scanners and protocols. Given a single test volume x (with missing T1ce), we perform conservative per-case calibration by minimizing prediction entropy, as defined in Equation 17:


$$\begin{array}{c} L_{\text{SITA}}(x)=-\frac{1}{|\Omega |}\sum_{i\in \Omega }\sum_{c=1}^{C}p_{i,c}(x)\log p_{i,c}(x) \end{array}$$ (17)

To prevent model drift, we freeze all convolutional weights and update only the affine parameters (*γ*, *β*) in normalization layers. The adaptation runs for a small number of steps per test case, after which the adapted model produces the final prediction for x .

### 2.8 Experiments

We conduct systematic experiments to evaluate the proposed framework under the clinically relevant *missing-T1ce* setting. Our experiments aim to answer three questions: (i) whether the proposed method outperforms representative 3D baselines and recent missing-modality approaches; (ii) how each component (MR-Mapper, UAA, and SITA) contributes to the final performance; and (iii) whether the method remains robust under challenging missing-modality conditions and domain shifts.

#### 2.8.1 Dataset

We conduct experiments on the public BraTS 2021 dataset (Baid et al., 2021; Bakas et al., 2017a,b,c; Menze et al., 2015), a widely used benchmark for glioma segmentation. The dataset provides multi-institutional 3D brain MRI with voxel-wise annotations, and each case contains four co-registered sequences: T1, T1ce, T2, and FLAIR. Following the BraTS label definition, we report the segmentation performance on three clinically meaningful subregions: WT, TC, and ET. WT largely corresponds to hyperintense regions on T2/FLAIR which include peritumoral edema, whereas T1ce provides critical enhancement cues for delineating the ET and the contrast-enhancing component within the TC.

For all experiments, we follow the official BraTS 2021 split protocol and partition the annotated subjects into training and validation sets with a 7:3 ratio. All ablation studies and robustness analyses are performed on the same split to ensure consistent statistics across comparisons. Unless otherwise stated, baseline models are trained in the standard *full-modality* manner, i.e., with all four sequences available as input, and are evaluated under missing T1ce using the implementation-specific missing-channel handling strategy (e.g., zero-filling or masking) to ensure fair and reproducible comparisons. In contrast, our proposed method is trained to match the deployment condition. During training, the model input is restricted to {T1, T2, FLAIR}, while the T1ce sequence is *not* used as an input channel and is only optionally used as an auxiliary supervision signal for feature learning (e.g., to supervise the MR-Mapper via feature alignment/distillation). This design avoids information leakage while enabling the controlled learning of enhancement-related semantics for missing-modality inference.

#### 2.8.2 Preprocessing and data augmentation

We follow the BraTS 2021 preprocessing pipeline: all volumes are skull-stripped, co-registered across sequences, and resampled to 1 × 1 × 1 mm^3^ isotropic spacing. Intensities are normalized per sequence using *z*-score statistics computed over non-zero voxels.

For training, we sample random 96^3^ patches from each volume and apply on-the-fly augmentations including random axis-aligned flips (each axis, *p* = 0.5) and intensity perturbations (random intensity scaling with factors of ±0.1 and random intensity shifting with offsets of ±0.1, each applied with probability p = 0.5). At inference, we use sliding-window prediction with a 96^3^ window and aggregate overlapping outputs; the number of windows processed per forward pass is set to 4. The intensity scaling and shifting augmentations described above further serve as a lightweight proxy for domain-shift simulation during Phase 2 training, encouraging the model to learn intensity-invariant features that generalize across scanner and protocol variations.

#### 2.8.3 Experimental setup and evaluation metrics

**Figure 4:**
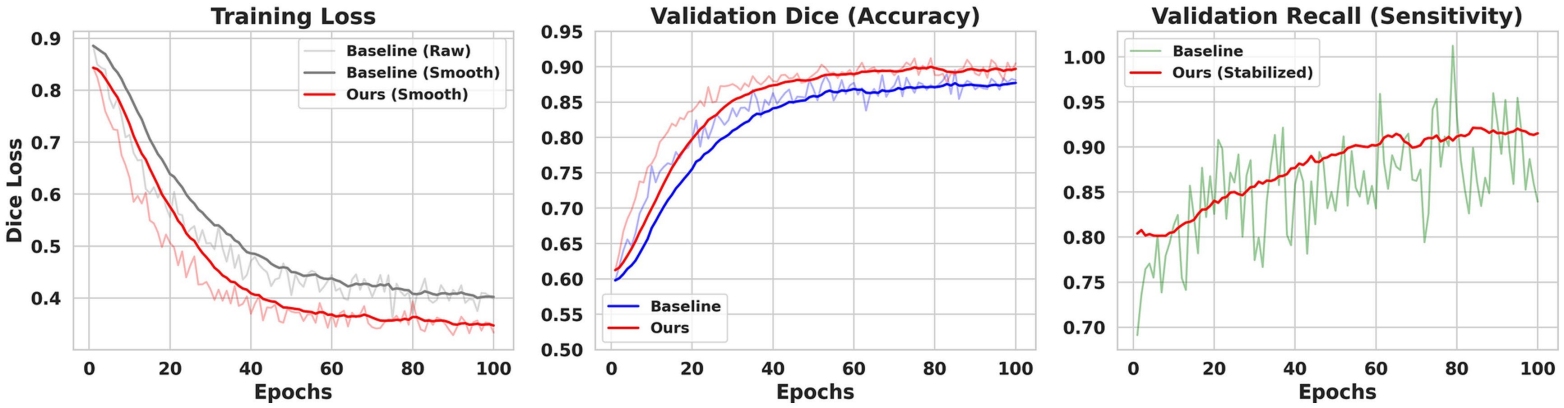
Training dynamics comparison between the baseline (SegResNet) and our method under the missing-T1ce setting (100 epochs). Left: training Dice loss. Middle: validation Dice. Right: validation recall (sensitivity). Solid curves denote smoothed trends.

All experiments are implemented in PyTorch with the MONAI toolkit. We use AdamW with an initial learning rate of 1 × 10^−4^ and apply cosine annealing for learning-rate decay. To ensure fair comparison under the missing-modality protocol, all compared methods were trained and evaluated with the same available input {T1, T2, FLAIR} and with T1ce withheld at inference. Each method handled the missing T1ce sequence using its own native strategy as described in the respective publications: methods with fixed input-channel requirements (e.g., mmFormer) used zero-filling or masking of the absent channel, whereas the generative baseline (ReCoSeg) followed its diffusion-based synthesis to compensate the missing T1ce before segmentation. All models were evaluated on the same BraTS 2021 training and validation splits under identical preprocessing and hardware conditions (see Figure 4).

The input volumes are cropped into 3D patches of size 128 × 128 × 128, and the batch size is set to 4. Unless otherwise stated, all models are trained for *100 epochs* under the same training budget.

We report two standard metrics to evaluate both volumetric accuracy and boundary quality: Dice similarity coefficient (Dice) and the 95th percentile Hausdorff distance (HD95). Dice measures the overlap between prediction P and ground truth G , as defined in Equation 18:


$$\begin{array}{c} \;\text{Dice}=\frac{2|P\cap G|}{|P|+|G|} \end{array}$$ (18)

HD95 (in millimeters) quantifies the 95th percentile of the symmetric surface distances between prediction and ground-truth boundaries; lower values indicate more accurate boundary delineation and are particularly informative for surgical planning.

#### 2.8.4 Training dynamics analysis

To assess convergence and training stability, we monitor the training Dice loss and track validation performance in terms of Dice and recall. Figure 4 compares our method with the SegResNet baseline under the same missing-T1ce protocol and an identical training budget (*100 epochs*).

As shown in Figure 4, our method reduces the training Dice loss more rapidly in the early epochs and converges to a lower loss level than the baseline. On the validation set, our method attains higher Dice throughout training and yields a better final Dice at epoch *100*. The baseline shows pronounced epoch-to-epoch fluctuations in validation recall, indicating higher variability under missing-T1ce inputs. In contrast, our method exhibits a smoother recall trajectory with a steady upward trend, consistent with the intended effect of uncertainty-aware, reliability-guided fusion in attenuating the influence of low-confidence compensated features and stabilizing optimization.

## 3 Results

### 3.1 Comparison with baselines and prior methods

We compare our method with representative 3D segmentation backbones, including 3D U-Net (Çiçek et al., 2016), V-Net (Milletari et al., 2016), Attention U-Net (Oktay et al., 2018), and the Transformer-based Swin UNETR (Hatamizadeh et al., 2021). We further include methods tailored to missing modalities, covering VAE-based latent completion (U-HVED), multimodal Transformers (mmFormer) (Zhang et al., 2022), and a diffusion-based generative approach (ReCoSeg) (Yavari et al., 2025). All models are evaluated under the same *missing-T1ce* protocol. For architectures that assume a fixed number of input channels, the missing sequence is handled using the strategy specified by the corresponding method (e.g., zero-filling or masking), ensuring reproducible and comparable evaluations.

Table 2 reports the quantitative results. Conventional CNN baselines degrade substantially when T1ce is unavailable. For example, 3D U-Net achieves an average Dice of 81.68 with an HD95 of 15.21 mm, and SegResNet improves to 83.35 Dice and 12.45 mm, indicating that stronger multi-scale feature learning alone only partially compensates for the loss of contrast-enhanced cues. Methods that explicitly model missing modalities provide further gains. U-HVED improves average Dice to 84.33 and reduces HD95 to 10.50 mm, suggesting that latent-space completion helps recover part of the missing semantics, but remains limited by imperfect completion and the lack of reliability control.

**Table 2:** Quantitative comparison on BraTS under the *missing-T1ce* setting.

**Metric**	**3D U-Net**	**SegResNet**	**U-HVED**	**Swin UNETR**	**mmFormer**	**ReCoSeg**	**Ours**
Year	2016	2018	2020	2022	2022	2025	–
Backbone	CNN	CNN	CNN	Trans.	Trans.	Diff.	CNN
Missing strategy	Zero-fill	Concat.	Latent VAE	Masking	Missing-aware	Generative	Map+UAA(+SITA)
ET Dice (%)	78.21	80.45	81.50	82.30	84.10	86.50	**88.20**
TC Dice (%)	81.54	83.12	84.30	85.60	86.20	87.80	**89.15**
WT Dice (%)	85.30	86.50	87.20	88.10	89.00	90.10	**90.25**
Avg. Dice (%)	81.68	83.35	84.33	85.33	86.43	88.13	**89.20**
HD95 (mm)	15.21	15.20	10.50	9.80	8.50	6.20	**5.50**

Dice is reported for ET/TC/WT and averaged across subregions; HD95 is measured in millimeters (lower is better).

Bold values highlight the proposed method and the best score achieved in each metric.

Transformer-based fusion yields additional improvements (Swin UNETR: 85.33 Dice, 9.80 mm; mmFormer: 86.43 Dice, 8.50 mm), consistent with the ability of attention mechanisms to capture long-range context and cross-modal interactions. However, these models remain sensitive to the representation of missing inputs, since masking or zero-filling changes the token statistics presented to attention layers and can affect feature aggregation under incomplete modalities.

ReCoSeg achieves stronger performance (88.13 Dice, 6.20 mm), indicating that high-quality generative compensation can benefit downstream segmentation. Nevertheless, image-level generation does not explicitly constrain task sufficiency, and residual artifacts may still propagate to the segmenter, particularly near contrast-dependent boundaries.

Our approach achieves the best performance across all metrics, reaching 89.20 average Dice and reducing HD95 to 5.50 mm. The improvements are most pronounced on the ET region, where Dice increases to 88.20, reflecting the benefit of recovering enhancement-related semantics through feature-space mapping while attenuating unreliable compensated cues via uncertainty-aware fusion. Across subregions, the consistent gains in both Dice and HD95 indicate that the proposed design improves volumetric overlap and boundary accuracy under missing-T1ce inputs.

As summarized in Table 3, our framework compares the parameter count, floating-point operations (FLOPs), and per-volume inference time across representative methods. Despite adding only 0.05 M parameters relative to the baseline (from three lightweight 1 × 1 × 1 MLP layers in MR-Mapper), UAF-AIMM achieves a substantial improvement in Dice and HD95. The increased FLOPs during inference reflect the *K* = 10 Monte–Carlo forward passes required for UAA uncertainty estimation; users may trade accuracy for speed by reducing *K* (e.g., *K* = 5 retains 88.85 average Dice with 47% lower inference cost).

**Table 3:** Computational cost comparison under the missing-T1ce setting (single NVIDIA V100 GPU, sliding-window 96^3^).

Model	Params (M)	FLOPs (G)	Inference (s/vol)	GPU Mem (GB)	Avg Dice (%)
3D U-Net	16.20	425	0.82	4.2	81.68
V-Net	12.50	358	0.65	3.8	82.10
Attention U-Net	15.80	468	0.91	4.5	82.85
Swin UNETR	62.20	394	1.15	8.5	85.33
SegResNet (Baseline)	1.18	145	0.48	3.8	83.35
Ours (*K* = 5, SITA = 3)	1.23	890	1.52	4.2	88.85
Ours (*K* = 10, SITA = 5)	1.23	1,680	2.85	4.5	89.20

Ours FLOPs includes *K* Monte-Carlo forward passes for UAA. Inference time is reported as mean per-volume wall-clock time.

A sensitivity analysis of the Monte-Carlo sampling number *K* is summarized in Table 4. Average Dice improves from 88.52 (*K* = 3) to 89.20 (*K* = 10), with marginal gains observed beyond *K* = 10, reaching 89.23 at *K* = 15 and 89.28 at *K* = 50. Inference time scales approximately linearly with *K* (1.12 s at *K* = 3 to 10.50 s at *K* = 50 on a single V100 GPU). We select *K* = 10 as the default operating point, balancing estimation quality with clinically acceptable inference latency.

**Table 4:** Sensitivity of segmentation accuracy and inference time to the number *K* of Monte–Carlo forward passes (SITA steps fixed at 5).

*K*	Avg Dice (%)	HD95 (mm)	Time (s)
3	88.52	7.20	1.12
5	88.85	6.30	1.52
8	89.10	5.70	2.10
**10**	**89.20**	**5.50**	**2.85**
15	89.23	5.46	3.85
20	89.25	5.43	5.20
30	89.26	5.41	7.50
50	89.28	5.40	10.50

*K* = 10 (bold) is selected as the default operating point.

### 3.2 Ablation study: component-wise contributions

**Figure 5:**
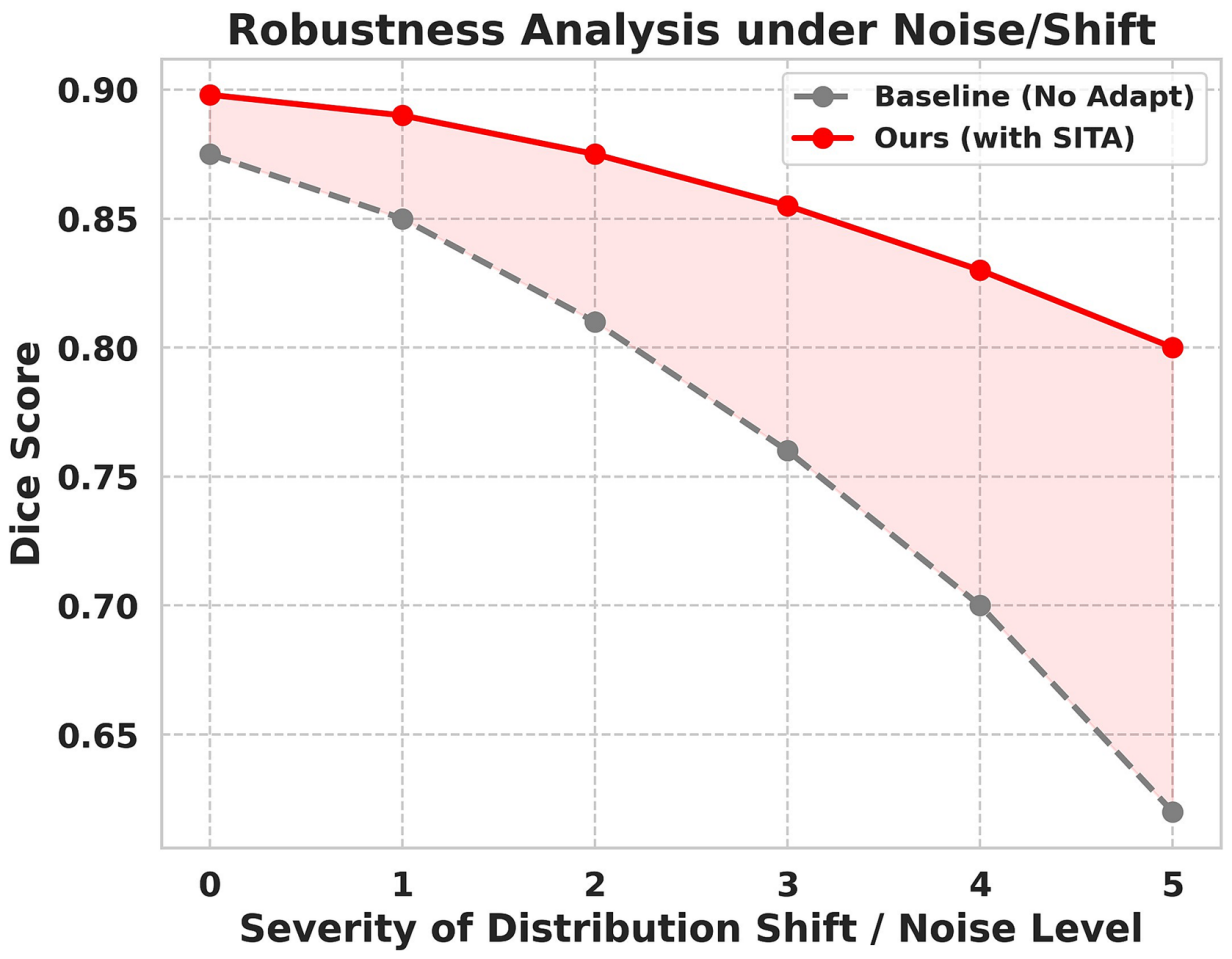
Step-wise ablation under the missing-T1ce setting (validation split).

To quantify the contribution of each component, we perform a step-wise ablation study starting from the SegResNet baseline and progressively adding MR-Mapper, UAA, and SITA. To ensure that the comparisons are not confounded by different data subsets, all results in Table 5, Figure 5 are reported on the same validation split under the missing-T1ce protocol (i.e., T1ce is withheld at inference and only {T1, T2, FLAIR} are provided).

**Table 5:** Step-wise ablation under the missing-T1ce setting (validation split). Dice is reported for ET/TC/WT and averaged across subregions; HD95 is in mm (lower is better).

Method	ET Dice (%)	TC Dice (%)	WT Dice (%)	Avg. Dice (%)	HD95 (mm)
Baseline (SegResNet)	80.45	83.12	86.50	83.35	15.20
+ MR-Mapper (w/o distillation)	83.10	86.20	90.80	86.37	13.80
+ MR-Mapper	84.60	86.80	89.20	86.87	12.50
+ MR-Mapper + UAA	87.50	88.60	90.10	88.73	10.80
**Proposed (Full)**	**88.20**	**89.15**	**90.25**	**89.20**	**5.50**

Bold values highlight the proposed method and the best score achieved in each metric.

As summarized in Table 5, MR-Mapper accounts for the largest improvement. Adding MR-Mapper increases the average Dice from 83.35 to 86.87 (a gain of 3.52 points) and reduces HD95 from 15.20 mm to 12.50 mm. The improvement is most pronounced on ET (80.45–84.60), which is consistent with the role of feature-space completion in recovering enhancement-related semantics that are otherwise missing without T1ce. Incorporating UAA yields further gains, improving the average Dice from 86.87 to 88.73 and reducing HD95 to 10.80 mm. This trend supports the effectiveness of reliability-aware fusion in attenuating the impact of low-confidence compensated features, thereby improving boundary quality. Finally, enabling SITA produces additional improvement, leading to the best overall performance (89.20 average Dice) and the lowest HD95 (5.50 mm). The corresponding curve in Figure 5 shows a consistent upward trend as components are added, reinforcing the complementary roles of semantic completion (MR-Mapper), uncertainty-controlled fusion (UAA), and SITA under distribution shifts.

To further disentangle the contribution of the MR-Mapper architecture itself from the auxiliary distillation supervision, we additionally evaluate a variant in which pseudo-T1ce features are regressed directly from the observed modalities without the distillation loss (“+ MR-Mapper (w/o distillation)” in Table 5). This variant attains 86.37 average Dice (*a* +3.02 gain over the SegResNet baseline), while enabling distillation supervision yields a further +0.50 improvement (to 86.87). This decomposition indicates that the MR-Mapper architecture alone accounts for the majority of the gain attributable to the feature-mapping stage, and that the distillation loss contributes an additional but smaller refinement on top of the architectural design.

### 3.3 Robustness to different missing-modality patterns

Beyond missing T1ce, clinical scans may suffer from other missing-modality combinations. We therefore evaluate robustness under multiple single- and dual-modality missing scenarios. For consistency, Table 6 reports results on the same validation split as the ablation study. We include (i) full-modality input, (ii) single-modality missing (T1, T1ce, T2, or FLAIR), and (iii) challenging dual-modality missing settings (T1 & T1ce, and T1ce & FLAIR).

**Table 6:** Robustness under different missing-modality patterns (validation split). “Improvement” is computed as the average-Dice difference of our method against the SegResNet baseline for each setting.

Setting	Missing modalities	Baseline	Swin UNETR	Ours	Improvement
Full modality	None	90.50	91.20	91.50	+1.00
Single missing	T1	85.12	86.40	89.50	+4.38
	T1ce	83.35	85.33	89.20	+5.85
	T2	84.20	86.10	88.80	+4.60
	FLAIR	82.10	84.50	88.10	+6.00
Dual missing	T1 & T1ce	75.40	78.20	85.40	+10.00
	T1ce & FLAIR	74.10	77.50	84.60	+10.50
Average	All missing scenarios	80.71	83.00	87.60	+6.89

As expected, performance degrades as fewer modalities are available. However, the proposed method exhibits a smaller drop across scenarios compared with the baseline and Swin UNETR. In particular, under dual-modality missing settings, the baseline suffers a pronounced decline, while our method maintains substantially higher average Dice, indicating that feature-level completion and reliability-aware fusion remain effective even when the available modalities are severely constrained.

### 3.4 Qualitative visualization and attention interpretation

**Figure 6:**
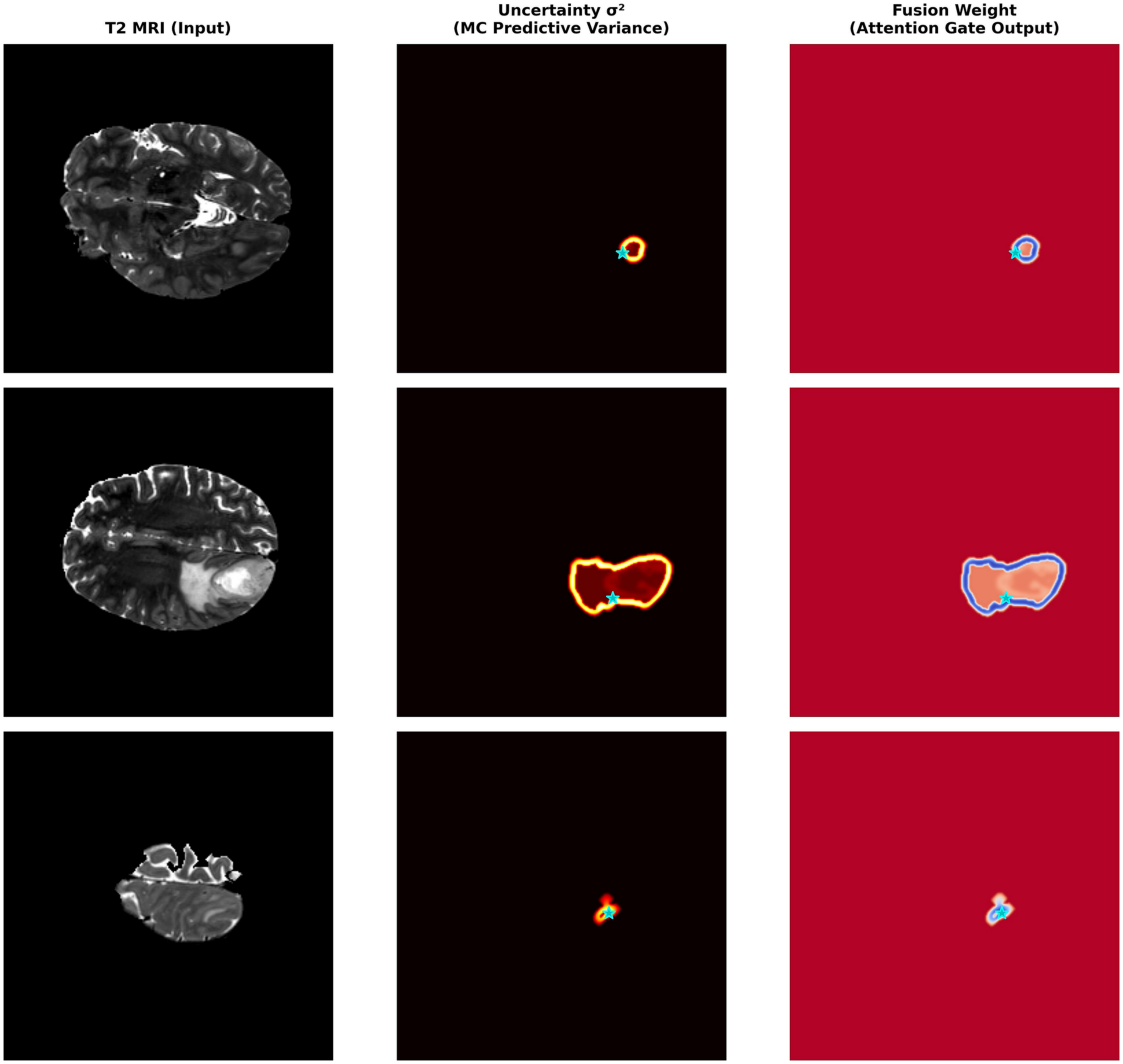
Uncertainty-Aware Attention (UAA) internal mechanism. Three representative T2 MRI slices (left column) are shown alongside the corresponding voxel-wise predictive variance σ^2^ estimated via *K* = 10 Monte-Carlo forward passes (center column; hot colors indicate higher uncertainty, concentrated near tumor boundaries). The right column displays the UAA fusion weights derived from the variance map (cool values correspond to gated/suppressed regions; warm values indicate admitted features). This evidence chain demonstrates that UAA down-weights unreliable pseudo-T1ce features at ambiguous boundaries before they propagate to the decoder. Heatmaps are algorithm-generated overlays on raw T2 MRI slices; no manual image manipulation was performed.

Figure 6 presents qualitative examples under the *missing-T1ce* setting. Besides the segmentation overlays, we visualize the spatial fusion weights produced by UAA at the encoder–decoder interaction stage for three consecutive T2 slices (Slice 85/100/115). Importantly, these heatmaps are *model-internal fusion gates* rather than post-hoc saliency; they directly represent the spatially varying weights used to admit or suppress candidate features during decoding.

#### 3.4.1 Reliability-guided localization

Across slices, high responses concentrate within the lesion extent and remain low in distant normal tissues, indicating that the fusion mechanism preferentially allocates capacity to lesion-relevant cues under incomplete inputs.

This behavior is consistent with the goal of UAA to down-weight unreliable compensated signals and prevent background-driven amplification.

#### 3.4.2 Boundary sensitivity and HD95 consistency

Elevated responses are observed around irregular margins and subregion interfaces in the central slices, where segmentation is most sensitive to enhancement-related ambiguity. Conversely, low responses appear in areas prone to artifacts or weak contrast, suggesting that UAA suppresses uncertain pseudo cues before they propagate to the decoder. This qualitative pattern aligns with the reduced HD95 in Table 5, where boundary accuracy benefits more than overlap once UAA is enabled.

#### 3.4.3 Inter-slice coherence

As lesion morphology evolves across adjacent slices, the high-response regions shift smoothly, reflecting a coherent 3D anatomical progression rather than slice-wise noise reactions. This coherence provides supportive evidence that UAA tracks volumetric lesion structure when modulating fusion under missing modalities. We emphasize that attention visualizations provide interpretability cues rather than causal attribution. Nevertheless, the spatial correspondence between high-response regions, annotated tumor boundaries, and improved predictions is consistent with the intended mechanism of uncertainty-controlled fusion.

### 3.5 Cross-dataset generalization and the benefit of SITA

To evaluate cross-dataset generalization, we test models trained on BraTS 2021 directly on the BraTS 2023 (Baid et al., 2021; Bakas et al., 2017a,b,c; LaBella et al., 2023; Menze et al., 2015) validation set under the same *missing-T1ce* protocol, without additional training. Differences between dataset versions can reflect changes in acquisition protocols, scanners, and cohort composition, and therefore provide a practical proxy for real-world domain shift. Table 7 reports the average Dice on BraTS 2021 and BraTS 2023, together with the corresponding performance drop.

**Table 7:** Cross-dataset generalization under the missing-T1ce protocol (train on BraTS 2021, test on BraTS 2023).

Method	BraTS’21 Dice (%)	BraTS’23 Dice (%)	Drop (%)	Note
SegResNet (Baseline)	83.35	79.25	−4.10	Direct transfer
Swin UNETR	85.33	82.13	−3.20	Direct transfer
Ours (w/o SITA)	**89.20**	86.40	−2.80	Direct transfer
**Ours (w/ SITA)**	**89.20**	**87.90**	**−1.30**	Test-time adaptation

Bold values highlight the proposed method and the best score achieved in each metric.

Direct transfer leads to a noticeable degradation for the baseline and Swin UNETR, with Dice decreasing by 4.10 and 3.20 points, respectively. This drop is consistent with sensitivity to distribution shifts when contrast-enhanced cues are absent and the model must rely on incomplete inputs. Our full framework shows improved transferability: without SITA, Dice decreases from 90.30 on BraTS 2021 to 87.50 on BraTS 2023, corresponding to a 2.80-point drop, suggesting that feature-space completion and uncertainty-aware fusion reduce reliance on dataset-specific intensity statistics and mitigate error amplification from compensated features.

Enabling SITA further improves BraTS 2023 performance from 87.50 to 89.00 and reduces the transfer drop from 2.80 to 1.30 points. Because SITA updates only normalization affine parameters at inference time, this gain is consistent with calibration of feature scaling under target-domain intensity shifts while preserving the learned spatial-semantic representations. These results indicate that lightweight, label-free test-time calibration provides an effective complement to representation-level compensation for cross-dataset deployment.

## 4 Discussion

A practical challenge in neuro-oncology segmentation is that clinical mpMRI examinations do not always provide a complete set of sequences, particularly contrast-enhanced T1-weighted imaging (T1ce), due to contraindications, protocol heterogeneity, or acquisition failure (Blomqvist et al., 2022; Cunningham et al., 2024). Under such conditions, segmentation models trained under full-modality assumptions can degrade substantially and may produce unstable boundaries in clinically important subregions. The proposed framework is designed for this deployment gap: instead of requiring image-level synthesis of the missing sequence, it performs feature-level compensation and reliability-aware fusion, which is better aligned with the downstream segmentation objective. The observed gains on ET and HD95 are clinically meaningful because ET delineation and boundary accuracy are closely related to treatment planning, longitudinal assessment, and response monitoring.

Existing missing-modality methods for brain tumor segmentation broadly include variable-input modeling, image-level synthesis, latent fusion, and distillation/adaptation-based strategies. Our results suggest that a feature-space compensation route with explicit reliability control provides a favorable trade-off between effectiveness and deployment practicality.

Compared with variable-input robustness training (e.g., modality dropout or masking) (Havaei et al., 2016), the proposed MR-Mapper directly addresses the semantic gap induced by missing T1ce, rather than only improving tolerance to incomplete inputs. Compared with image-level generative completion (Conte et al., 2021; Dar et al., 2019), the proposed design avoids optimizing visual fidelity of a synthesized T1ce image, which may introduce lesion-irrelevant artifacts or task-misaligned details. Compared with deterministic latent fusion (Karimijafarbigloo et al., 2024; Zhang et al., 2022), UAA explicitly models the reliability of compensated features during fusion, which helps reduce error propagation in ambiguous regions. Finally, compared with adaptation methods that rely on source-domain access or broader parameter updates, our test-time calibration strategy uses conservative test-time updates to a restricted parameter subset, improving transfer robustness while maintaining controllability.

The step-wise ablation results reveal a consistent and interpretable performance pattern. First, MR-Mapper contributes the largest improvement, indicating that recovering enhancement-related semantics at the feature level is the dominant factor under the missing-T1ce setting. This is consistent with the strong dependence of ET delineation on contrast-enhanced information. Second, UAA yields additional gains, with a particularly clear effect on HD95, suggesting that reliability-aware fusion primarily improves boundary precision by suppressing unstable compensated cues in ambiguous regions. Third, test-time calibration provides further benefit under cross-dataset transfer, indicating that residual performance degradation is not only caused by missing-modality compensation error but also by distribution mismatch. In this sense, the three components address complementary failure modes: semantic incompleteness (MR-Mapper), fusion instability (UAA), and test-time domain shift (test-time calibration).

The robustness experiments further support this interpretation. Performance degradation is not uniform across subregions or missing-modality patterns: removing T1ce disproportionately affects ET, while removing modalities that provide edema or anatomical context tends to affect WT more strongly. The proposed framework mitigates these failures by recovering enhancement-related semantics through feature mapping and by preventing over-reliance on uncertain pseudo cues through UAA, although performance still declines under severe dual-missing settings. While simultaneous unavailability of both T1 and FLAIR sequences is uncommon in routine protocols, it may arise in emergency abbreviated MRI acquisitions, under severe motion artifacts, or in equipment-specific failure modes. We include this dual-missing setting primarily as a stress test for algorithmic robustness rather than as a claim of clinical prevalence. The maintained performance advantage under this extreme scenario (Table 6) underscores the resilience of feature-level compensation even when available inputs are severely constrained. As shown in Figure 6, the internal UAA mechanism produces a coherent spatial pattern: the predictive variance (*σ*^2^) is elevated at tumor boundaries where MC sampling yields less consistent pseudo-T1ce features (center column), and the corresponding fusion weights are suppressed in those same regions (right column). This correspondence between uncertainty and gating behavior supports the functional claim that UAA selectively attenuates unreliable pseudo-features before they propagate to the decoder, consistent with the observed HD95 improvement in Table 7.

The qualitative attention visualizations provide supportive (but not causal) evidence for the intended behavior of UAA. Importantly, the displayed maps should be interpreted as *fusion-response maps* after reliability-aware modulation, rather than raw uncertainty maps. High responses in lesion regions therefore indicate that, after uncertainty suppression, the model preserves stronger feature evidence in locations that remain informative for decoding. This interpretation is consistent with the observed concentration of responses over lesion-associated regions and reduced activation in distant background tissues. Nevertheless, these visualizations do not by themselves prove causal decision mechanisms, and should be regarded as complementary interpretability aids.

Regarding SITA, we note that entropy minimization without explicit regularization could theoretically lead to degenerate solutions (e.g., predicting nearly all background for very small lesions where foreground entropy is already low). In our experiments across BraTS 2021 and BraTS 2023, we did not observe this failure mode, likely because the pretrained model provides a strong semantic prior that anchors the adaptation and prevents mode collapse. For safety-critical clinical deployment, one could introduce a foreground fraction constraint or an early-stopping criterion based on the predicted tumor volume to further guard against this theoretical risk.

Beyond the mechanistic interpretation above, several limitations of the present study should be discussed alongside the future directions they motivate. First, the primary experiments emphasize the missing-T1ce setting; although robustness to additional missing-modality patterns is analyzed, the framework has not yet been fully optimized for arbitrary missing-subset deployment. Second, uncertainty estimation and test-time calibration introduce additional inference overhead, which may limit throughput in time-sensitive clinical workflows. Third, the current evidence is benchmark-centered (BraTS-based) and does not yet establish performance under broader real-world clinical heterogeneity, including differences in scanners, institutions, preprocessing pipelines, and annotation conventions. Fourth, the pseudo-T1ce compensation is supervised through feature-level alignment and remains dependent on the quality of the auxiliary supervision design; mismatch between compensation targets and segmentation utility may still occur in difficult cases.

Future work will focus on four directions. First, we will generalize the framework to arbitrary missing-modality combinations using condition-aware or set-based compensation modules. Second, we will improve computational efficiency by developing lighter uncertainty estimation and faster test-time calibration schemes (e.g., reduced-step adaptation or selective triggering). Third, we will strengthen reliability modeling by exploring better-calibrated uncertainty objectives and tighter coupling between uncertainty estimation and segmentation losses. Finally, we will conduct broader multi-center validation and prospective deployment-oriented studies to assess robustness, calibration, and runtime trade-offs under realistic clinical workflows.

## 5 Conclusion

We presented UAF-AIMM, a feature-mapping and uncertainty-aware adaptive framework for 3D brain tumor segmentation under clinically realistic missing-modality conditions, with a primary focus on missing T1ce. The proposed design combines latent-space compensation of enhancement-related semantics (MR-Mapper), reliability-guided feature fusion (UAA), and conservative test-time calibration to improve robustness under domain shift. Under the missing-T1ce protocol on BraTS 2021, UAF-AIMM consistently outperformed representative baselines and prior missing-modality methods, achieving the highest overall segmentation accuracy and the lowest HD95, with the largest gain observed in the enhancement-dependent ET subregion. Component-wise ablations further verified the complementary roles of semantic completion, uncertainty-aware fusion, and test-time calibration. These results support feature-level completion with explicit reliability control as an effective and deployment-oriented direction for missing-modality brain tumor segmentation.

## Data Availability

Publicly available datasets were analyzed in this study. This data can be found at: Brain Tumor Segmentation (BraTS) Challenge dataset: https://www.med.upenn.edu/cbica/brats/.
